# Schumpeter meets Goldilocks: the scarring effects of firm destruction

**DOI:** 10.1007/s13209-023-00273-3

**Published:** 2023-03-08

**Authors:** Beatriz González, Enrique Moral-Benito, Isabel Soler

**Affiliations:** 1 Banco de España, Madrid, Spain; 2 European University Institute, Fiesole, Italy

**Keywords:** Firm dynamics, Firm exit, Productivity, COVID-19, E22, G33, M21, O47

## Abstract

This paper uncovers an inverted U-shaped relationship between firm exit and total factor productivity (TFP) growth using Spanish data. At low levels of firm exit, Schumpeterian cleansing effects dominate and the effect of firm destruction on TFP is positive, but when exit rates are very high, this effect turns negative. In order to rationalize this finding, we build on Asturias et al. (Firm entry and exit and aggregate growth, Technical report, National Bureau of Economic Research, 2017) and develop a model of firm dynamics with exit spillovers calibrated to match the nonlinearity found in the data. This reduced-form spillover captures amplification effects from very high destruction rates that might force viable firms to exit, for example, due to disruptions in the production network and a generalized contraction in credit supply. Armed with the calibrated model, we perform counterfactual scenarios depending on the severity of the shock to firm’s outcomes. We find that when the shock is mild and firm destruction rates at impact are similar to those observed during the Global Financial Crisis (GFC), TFP growth increases, and the recovery is faster. However, when the shock is severe and firm exit is well above that of the GFC, TFP growth decreases, since high-efficiency firms are forced out of the market, which makes the recovery much slower.

## Introduction

The COVID-19 pandemic brought an unprecedented economic disruption, halting for months most of the developed economies and forcing firms to close and/or operate below capacity due to social distancing measures. When the most acute phase of the pandemic was over, supply chain disruptions and turmoil in energy markets, recently aggravated by the war in Ukraine, originated an increase in firms’ operating costs not seen over the last few decades. In this context, there is a growing concern about the risk of a wave of firm defaults, with far-reaching implications for the evolution of aggregate TFP.^1^

More generally, the effect of firm exit on aggregate TFP is ambiguous. On the one hand, if it is low-productivity firms exiting, it might be the case that resources are allocated towards more productive firms or products, hence increasing aggregate productivity (Caballero and Hammour 1994). On the other hand, it may be the case that the magnitude of the shock is large enough to also hurt the position of productive and viable firms, forcing them to exit. Furthermore, a high exit rate could have negative effects even on other firms through input–output linkages, and also bankruptcies might make banks tighten their credit supply, amplifying the negative effects of the shock. These amplification effects might make productive firms exit and deter productive firms from entering (Hallward-Driemeier and Rijkers 2013; Sedláček 2020). Using data for Spain, we contribute to this strand of research by showing that the relationship between firm exit rates and aggregate TFP is an inverted U-shaped, and we show in a stylized model that features this nonlinearity the implications for firm dynamics and aggregate TFP. Understanding these patterns is of paramount importance in the current juncture, especially for an economy such as the Spanish one, characterized by its sluggish productivity growth over the last two decades.

First, we use firm-level data for Spanish non-financial firms from 2000 to 2018 to document the patterns of productivity growth in the last decades and to better understand the relationship between productivity growth and exit rates in Spain. The data comes from Central de Balances Integrada (CBI), which includes the quasi-universe of Spanish firms. One caveat of this dataset (as is the case with many business registries) is that sometimes firms disappear and reappear in the dataset, without actually exiting the economy. In order to be able to capture the actual exit of firms, we match CBI with micro-data on exit from the Spanish National Statistical Agency (Directorio Central de Empresas—DIRCE) that combines data from the tax agencies, social security filings and the mercantile registry to create an exit indicator at the firm level. We follow Foster et al. (2001) in their methodology to decompose aggregate TFP growth into four different components: entry, exit, reallocation and within-firm growth. As it has been widely documented, Spain had a boom period until 2007 characterized by the increase in misallocation and the decrease in TFP (see, for instance, García-Santana et al. 2020; Díaz and Franjo 2016 or Gopinath et al. 2017 for different explanations of this phenomenon). During the burst period (2008–2013), Spain suffered a deep crisis with TFP decreasing even further. In the last years of our sample (2013–2018), which we call the recovery period, TFP increased by 2.7%. One characteristic that is common to the three sub-periods is the positive (and modest) contribution of the exit margin to TFP growth, and this contribution seems to increase in times of crisis when exit rates are larger. This suggests that the ‘cleansing effects’ of exit are dominant in the aggregate and are thus a driver of TFP growth in Spain.^2^

Second, we study further this relationship between exit rates and TFP growth, and we document a new empirical fact. We show that, at the sector level, exit rates are nonlinearly related to future TFP growth. More concretely, we uncover an inverted U-shaped relationship between firm exit rates and TFP growth. That is, when exit rates are low, an increase in exit rates is associated with higher TFP growth, which seems to point at the ‘cleansing effects’ previously discussed: less productive firms exit the economy and hence resources shift towards high-productivity firms, increasing aggregate TFP growth. However, when exit rates are high, an increase in firm destruction is associated with a decrease in TFP, which seems to point at the ‘scarring effects’ of crises that induce large increases in exit. These results are robust to including controls, sector and year fixed effects, and running output-weighted regressions.

Third, we build a firm dynamics model à la Hopenhayn with growth building on Asturias et al. (2017) that is able to match the nonlinearity found in the data. In the model, firms face perfect competition, they are heterogeneous in their marginal efficiencies to produce, and they enter and exit endogenously, although there are also exogenous exits. The growth rate of the economy is exogenous, but the level underlying this balanced growth path (BGP) is endogenous and depends on three types of distortions: (a) potential entrants face firm entry costs; (b) these new firms face barriers that prevent them from adopting the most efficient technology; (c) there is a fixed continuation cost that firms need to pay every period. We are going to focus on distortion (c); that is, we think of the COVID-19 shock as a transitory increase in the cost of operation for firms, so all firms’ profits fall. This shock is especially hurtful for small and low-efficiency firms since this fixed cost bears a larger weight on these firms’ profits. In order to rationalize our empirical finding through the lens of the model, we add a fourth distortion: the exogenous exit rate depends on the level of the fixed cost for operation, which implies that the higher the fixed cost of operation is, the higher the exogenous exit rate is. We assume an exponential functional form for this relationship, and we calibrate the parameters to match the nonlinearity we observe in the data, together with other targets of the Spanish economy.

Fourth, we perform different counterfactual scenarios with the calibrated model. The scenarios depend on different assumptions on the impact of the COVID-19 shock and the subsequent developments (supply bottlenecks, surge in inflation, etc.) on exit rates. In particular, we make assumptions about two alternative scenarios for exit rates that we label as a mild and severe shock based on the increase in the cost of operation that delivers different exit rates in the model. In the case of the mild shock, we assume that the increase in the operation cost is moderate so that only those firms that were at risk of non-viability in 2020 exit. This would imply an increase in exit rates at impact that is close to that of the Great Recession. In this scenario, the increase in exit rates is accompanied by an increase in aggregate TFP: the increase in the cost of operation forces low-efficiency firms to exit; hence, resources are allocated towards higher-efficiency firms. In the case of the severe shock, we assume that all firms with a ratio of net worth to equity below 0.5 in 2020 exit (in Spain this is the condition to legally file for bankruptcy), and that this increase in exit rate is persistent. This shock is larger than the mild shock: it would imply an increase in exit rates at impact that is nearly four times as large as that of the most affected sector during the Great Recession, real estate. This large increase in exit rates increases significantly exit spillovers, which means that it is not only low-efficiency firms that exit, but also high-efficiency firms are forced out of the market, so aggregate TFP growth decreases. In the longer run, TFP decreases even further in the case of the severe scenario before converging back to the trend and the recovery is much slower, mainly due to the missing mass of firms that takes a long time to be substituted in the economy, especially those that are high-efficiency.

The rest of the paper is organized as follows. Section 2 presents the data. Section 3.1 documents the TFP decomposition in Spain with a special focus on the exit rate term. Section 3.2 analyses the effect of the exit rates on the TFP at a sector level, uncovering a nonlinear relationship. Section 4 presents the model à la Hopenhayn with endogenous and exogenous exit, which is calibrated with the Spanish economic data in Sect. 5. Section 6 presents the counterfactual exercises performed with the model, and Sect. 7 concludes.

## Data

Our analysis relies on the Integrated Central Balance Sheet Data Office (CBI), a detailed administrative dataset from the Bank of Spain obtained from obligatory filings of annual accounts in mercantile registries. The CBI is an unbalanced panel that includes firm-level data from 2000 to 2018, and it covers almost all Spanish firms; therefore, it is a representative sample. Each year, every firm reports its number of workers, its quantity of tangible capital and output, the value-added generated, and its ratio of leverage, among other variables. Using these data, we compute the total factor productivity (TFP) à la Wooldridge (2009). Appendix 1 provides a brief description of all the used variables and the cleaning process performed. Table 1 reports the descriptive statistics of the full sample.

**Table 1:** **Summary statistics**

	Obs	Mean	p50	p95	sd
Employment	6,951,552	15	4	35	249
Tangible capital	6,951,552	368	48	1,692	1,070
Value added	6,951,552	408	114	1,524	1,075
Output	6,951,508	1,445	339	5,766	3,823
Leverage	6,944,962	0.23	0.098	0.78	0.35
TFP	6,951,552	7.2	3.8	23	11

Source: CBI. Employment is the average number of employees. All nominal variables are in thousands of euros in 2015. Leverage is the ratio of debt to total assets. TFP is in levels. For more information about the cleaning procedure, see Appendix 1

**Fig. 1:**
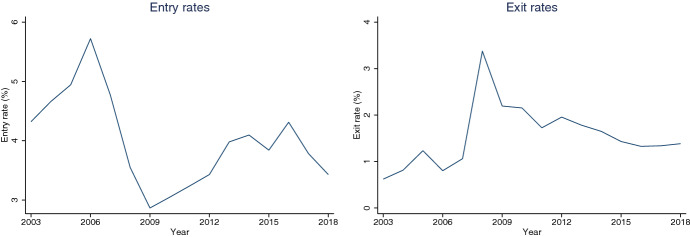
Entry and exit rates in Spain: 2000–2018. Notes: Source: CBI and DIRCE. The left-hand side figure shows the entry rate in percentage by year. The right-hand side figure shows the exit rate in percentage by year

The year of the constitution of each firm is provided in CBI; however, the year of exit is much harder to obtain from this dataset, especially given the attrition rates. Hence, to obtain the exit of a firm, we merge our data with micro-data from the Directorio Central de Empresas (DIRCE), a dataset from the National Statistical Institute (INE). DIRCE, combining different data sources (tax data, mercantile registry and social security instances) creates a flag for entry and exit for each firm the year when this event occurs.^3^ During the Great Recession, all sectors experienced an increase in exit rates: the median sectoral exit rate increased 1.6% points and the greatest increase in exit rate was that of the real estate sector, with a 3.9% points increase in exit rate in 2008. The aggregate exit rate increased 2.4% points (see Fig. 1).

**Table 2:** FHK decomposition for Spain

Year	TFP growth	Within	Reallocation	Entry	Exit
2003–2008	$-$0.0493	$-$0.1355	0.1022	$-$0.0172	0.0012
2008–2013	$-$0.0245	$-$0.1640	0.1462	$-$0.0124	0.0056
2013–2018	0.0271	0.0059	0.0268	$-$0.0094	0.0039

Source: CBI and DIRCE. This table shows our calculations of the FHK decomposition with Spanish data, according to the equation 3.1. For this calculus, we consider three different groups in each period: continuers, entrants and exiters. For example, in 2003–2008, continuers are firms that have observations in 2003 and 2008, entrants are firms that have an observation in 2008 and have started in the market before 2008 but after 2003, and exiters are firms that have observations in 2003 but exit before 2008; the rest of the firms that do not meet these conditions are not considered

## Empirical findings

### FHK decompositions

One of the most widely used decompositions is that of Foster et al. (2001) (FHK henceforth), which takes into account not only changes in the productivity distribution of incumbents but also changes due to the exit-entry margin. We start performing the FHK decomposition on Spanish firms for three different 5-year sub-periods in the sample: 2003–2008, 2008–2013 and 2013–2018. TFP growth of industry *i* at time *t* is defined as

$$\begin{aligned} \Delta \log Z_{it}=\sum _{e\in i}s_{eit}\log (z_{eit})-\sum _{e\in i}s_{ei,t-1}\log (z_{ei,t-1}) \end{aligned}$$

where $Z_{it}$ is the TFP of industry *i* at time *t*, $z_{eit}$ is the firm *e*’s TFP, $s_{eit}$ is the share of firm *e*’s output in industry *i*. FHK decomposes TFP growth in the within term, the reallocation term, the entry term and the exit term as follows.

$$\begin{aligned} \Delta \log Z_{it}&=\underbrace{\sum _{e\in C_{it}}s_{ei,t-1}\Delta \log (z_{eit})}_{\text {within term}}+\underbrace{\sum _{e\in C_{it}}\Delta s_{eit}(\log (z_{eit})-\log (Z_{i,t-1}))}_{\text {reallocation term}}\\&\quad + \underbrace{\sum _{e\in N_{it}}s_{eit}(\log (z_{eit})-\log (Z_{i,t-1}))}_{\text {entry term}}\\&\quad +\underbrace{\sum _{e\in X_{it}}s_{ei,t-1}(\log (z_{ei,t-1})-\log (Z_{i,t-1}))}_{\text {exit term}}, \end{aligned}$$

where $C_{it}$ is the set of continuing firms in the industry *i* at time *t*, $N_{it}$ is the set of entrants and $X_{it}$ is the set of exiters.

Table 2 shows the FHK decomposition to firm-level data for Spain. As other papers in the literature have shown (see for instance Díaz and Franjo 2016; García-Santana et al. 2020; Fu and Moral Benito 2018 or Gopinath et al. 2017), the period of the boom in Spain (2003–2008) is characterized by negative TFP growth, with the within term contributing the most to the negative TFP growth. During the burst (2008–2013), TFP growth is still negative, but there is an increase in the positive impact of the reallocation and the exit term. During the recovery period (2013–2018), TFP growth turns positive, especially because the within term becomes positive. When we look at the whole period (2003–2018), we can observe that the two components that remain always positive are the reallocation term and the exit term. The patterns observed here are similar to those of Riley et al. (2015) for the UK, who also find a negative contribution of the within term and the entry term, but a positive impact of the exit term and the reallocation term. However, the positive contribution of exit is not large enough to counteract the negative contribution of entry.

This evidence points to a mal-functioning creative destruction process in Spain since entry is contributing negatively to TFP growth and the positive contribution of exit is not enough to counteract this negative impact. It is noteworthy to realize how the contribution of exit increases during the burst, and decreases (slightly) during the recovery. This could be due to differences in the productivity of exiters, or due to changes in the share of exiters. Looking at Table 3, it turns out that the difference in productivity between stayers and exiters has been increasing during the whole sample, but, while the share of exiters was especially large during the burst, it was much lower during the recovery period.

**Table 3:** Exit shares versus productivity of exiters

Year	Share exit	Productivity
		continuers-exiters
2003	0.011	0.270
2008	0.018	0.487
2013	0.008	0.639

Source: CBI and DIRCE. This table shows the share of the exiters in each period and the difference in the productivity of continuers and exiters in the indicated year. We consider continuers the firms that are observed in the database after 5 years after the indicated year, while exiters are the firms that exit at some point in the 5 years after the indicated year

According to this evidence, it seems that the exit margin positive contribution is low but relevant, especially during bursts when exit rates are larger, which is supportive of the well-known ’cleansing effects’ of crises (Caballero and Hammour 1994; Osotimehin and Pappadà 2017). But is this always the case? An increase in exit rates might be beneficial for productivity growth since it forces low-productivity firms out of the market. However, if the shock is too large, it might trigger additional channels that might affect negatively TFP. These channels might encompass supply–demand disruptions and macroprudential risks that make banks cut financing to viable businesses. All of these channels would increase exit rates while decreasing TFP. Hence, the relationship between exit rates and TFP growth might be nonlinear, an issue we explore in the next section.

### Exit rates and TFP growth: uncovering a new nonlinearity

We next turn to use sector-level data to test whether the relationship between exit rates and TFP growth is nonlinear. Our sector-year level data features a mean exit rate of 1.3%, with a standard deviation of 9.8.^4^ In order to test for this nonlinearity, we regress the TFP output weighted growth (in 5 years) of each sector on the exit share and the square of the exit share in that economic sector. The general specification is

1 $$\begin{aligned} \vartriangle y_{it}=\beta _{0}+\beta _{1}exit{}_{it}+\beta _{2}exit{}_{it}^{2}+\beta _{3}C_{it}+\delta _{i}+\mu _{t}+v_{it} \end{aligned}$$

where $\vartriangle y_{it}$ is the TFP growth in the industry *i* from year $t-1$ to year $t+5$, here the TFP is weighted by each firm output. $exit_{it}$ is the main variable of interest measured as the exit rate in the sector *i* and year *t*. $C_{it}$ is a vector of controls: the lagged TFP weighted by the output, the lagged TFP unweighted and the rate of entry. And finally, fixed effects of sector and year are included $(\delta _{i},\mu _{t})$ In Table 4, different versions of this specification are shown.

**Table 4:** Regressions. Nonlinear effect of the exit rate on the TFP growth

	(1)	(2)	(3)	(4)	(5)
	TFP growth	TFP growth	TFP growth	TFP growth	TFP growth
	(5 years)	(5 years)	(5 years)	(5 years)	(5 years)
Exit rate	6.08***	8.64**	5.62**	8.12**	2.95*
	(1.96)	(3.24)	(1.96)	(3.22)	(1.44)
Exit rate$^2$	$-$99.48**	$-$197.79**	$-$89.59**	$-$182.88**	$-$36.66*
	(36.62)	(67.42)	(34.96)	(60.02)	(17.65)
Observations	712	712	711	711	711
*R*-squared	0.06	0.07	0.08	0.22	0.62
Sector FE	No	No	No	No	Yes
Year FE	No	No	No	No	Yes
Controls	No	No	Yes	Yes	Yes
Weighted	No	Yes	No	Yes	No

This table shows 5 different regressions where the dependent variable is the TFP growth in each sector-year group, and the independent variables of interest are the exit rate of each sector-year group and the squared exit rate, for a more detailed explanation of the specification, look at Eq. (1). Different versions of the main specification are included in order to assure the robustness of the result. Considered controls are the lagged TFP weighted by the output, the lagged TFP unweighted and the rate of entry. When the regression is weighted, it is weighted by the output of each year-sector. Standard errors are denoted in the parentheses. Asterisks denote statistically significant coefficients at 10% (*), 5% (**) and 1% (***), respectively

In column 1, we observe that the coefficient of the exit rate is positive, whereas the coefficient of the exit rate squared is negative (and both of them are significant). This means that when the exit rate is low then the effect of the exit is pro-competitive, however, when the exit rate is too high, then the TFP declines. Furthermore, this nonlinearity is still present when including the output weights of the sectors (column 2). We also find the described inverted U-shape relationship in columns 3, 4 and 5, where we include the lagged TFP weighted by the output, the lagged TFP unweighted and the rate of entry as controls. In column 4, the regression is weighted by the output and in column 5 the sector and year fixed effect are included to control for the business cycle and sector-specific characteristics. All specifications support a nonlinearity present in the data and predict negative TFP growths when exit rates are larger than 3.2-$-$5.7%, depending on the specification.^5^

In conclusion, this evidence points at what we call the ‘Goldilocks effect’ of the exit rate in the TFP, that is, for increases in exit rate to have ‘cleansing effects’, we need that the levels of exit rates are not too high.

## Model

Our baseline model is based on Asturias et al. (2017), who build a simple dynamic general equilibrium model of firm entry and exit based on Hopenhayn (1992). The model features a balanced growth path (BGP), where aggregate variables grow at an exogenous rate. Time is discrete. There is a continuum of firms in a closed economy. Firms are heterogeneous in their marginal efficiencies to produce, and all firms produce the same good in competitive markets. Entry and exit in the economy is endogenous, but there exists exogenous exit too. Although the growth rate of the economy is exogenous, the *level* underlying this BGP is endogenous and depends on distortions. In Asturias et al. (2017), there are three types of distortions: (a) potential entrants face firm entry costs; (b) these new firms face barriers that prevent them from adopting the most efficient technology; (c) there is a fixed continuation cost that firms need to pay every period. The distribution from which potential entrants draw their efficiencies exogenously improves each period; and the efficiency of existing firms improves both through an exogenous process and spillovers from the rest of the economy. We extend the model with a fourth distortion, which is going to capture in a reduced-form way the nonlinearity found in the empirical evidence discussed in the previous section: the exogenous exit rate depends on the level of the fixed cost for operation, which implies that the higher the fixed cost of operation is, the higher exogenous exit is. We assume an exponential functional form for this relationship, and we calibrate the parameters to match the nonlinearity we observe in the data. One key distinction in linking this model to the data is that firm efficiency is not the same as productivity, so we compute firm level productivity in the model following closely Asturias et al. (2017).^6^

### Household

There is a representative household that supplies labour $L_{t}$ inelastically to firms. She receives the dividends $D_{t}$ paid by firms net of the entry costs needed to finance the firm, consumes $C_{t}$ and saves in bond holdings $B_{t}$ maximizing utility according to the following maximization problem, where $\beta$ is the discount factor of the household:

2 $$\begin{aligned} {\max _{C_{t,}B_{t}}}{ \sum _{t=0}^{\infty }\beta ^{t}\log C_{t}}. \end{aligned}$$

s.t.

$$\begin{aligned}&P_{t}C_{t}+q_{t+1}B_{t+1}=w_{t}L_{t}+B_{t}+D_{t}\\&C_{t>0;}\ B>{ {B}};\ B_{0}\ given. \end{aligned}$$

### Firms

As previously explained, there is a continuum of firms producing the same final good. Note that all firms are owned by the household, so any inflow of funds to firms is financed by the household, entitling her to the dividends of the firms. The problem solved by incumbent firms and firms entering is the following.

**Incumbents**

Firms have access to a decreasing return to scale technology to operate

3 $$\begin{aligned} y=xl^{\alpha },\ \ \ 0<{\alpha <1;} \end{aligned}$$

where *l* is the amount of labour used to produce, and *x* is firms’ efficiency. The profit maximization problem is static and solves this maximization problem, where *w* are the wages paid:

4 $$\begin{aligned} \pi _{t}(x)={\max _{l}}\ \ xl^{\alpha }-wl. \end{aligned}$$

The dynamic problem of the firm is

5 $$\begin{aligned} V_{t}(x)={\max }{\{\pi _{t}(x)-f_{t}+q_{t+1}(1-{\delta (f_{t}))}V_{t+1}(xg_{c,t+1}),0\}}. \end{aligned}$$

Several terms deserve an explanation here. Firms need to pay a fixed cost of operation each period, $f_{t}$, so that dividends distributed to the household each period are $d_{t}(x)=\pi _{t}(x)-f_{t}$. The fixed cost of operation is assumed to grow at rate $g_{e}$. The stochastic discount factor coincides in equilibrium with the price of the one-period bond, $q_{t+1}$. The main modelling difference with Asturias et al. (2017) is the exogenous exit term ${\delta }:$ while in their paper it is a constant, here it is an increasing function of the fixed cost of operation $f_{t}$. Further below in this section, we provide further details about the choice of functional form for this equation and the rationale for it. The efficiency growth factor is characterized by

6 $$\begin{aligned} g_{ct}=\bar{g}g_{t}^{{\varepsilon }}, \end{aligned}$$

where $\bar{g}$ is a constant, $g_{t}$ is the growth factor from $t-1$ to *t* of the unweighted mean efficiency of all firms that operate in each period. The degree of spillovers from the aggregate economy to the firm is given by $\varepsilon$.^7^ As Eq. (5) states, a firm continues to operate only if the continuation value is greater than 0. Since the continuation value is increasing in *x*, one can show that there exists a $\hat{x}$ such that

7 $$\begin{aligned} V_{t}(\hat{x_{t}})=0. \end{aligned}$$

Firms decide to continue operating if $x>\hat{x_{t}}$, and they decide to exit endogenously otherwise.

**Entrants**

Each period, the mass of potential entrants can decide to pay a fixed cost ${\kappa _{t}}>0$ to draw a marginal efficiency $x_{t}$ from the Pareto distribution

8 $$\begin{aligned} F_{t}(x)=1-\left( \frac{\varphi _{t}x}{g_{e}^{t}}\right) ^{-\gamma }\ \ \ x\ge g_{e}^{t}/\varphi _{t} \end{aligned}$$

The mean of this distribution is proportional to $g_{e}^{t}/\varphi _{t}$, so it grows at an exogenous rate $g_{e}>1$. This implies that each cohort that enters has higher average efficiency than the previous one, crowding out low-efficiency firms from previous cohorts.^8^ The parameter $\varphi _{t}$ characterizes the barriers to technology adoption: if $\varphi _{t}>1$, the distribution is stochastically dominated by the frontier distribution, so the greater increasing the barriers to technology adoption lowers the mean efficiency of potential entrants.

Once the firm observes the draw of *x*, the potential entrant chooses whether to establish the firm with that efficiency (*successful entrant*) or not (*failed entrant*). Once the entrant establishes the firm, she faces the problem of an incumbent firm. The mass of entrants, ${\mu }$, is given by the free entry condition, which states that the expected value of entering equals the entry cost,

9 $$\begin{aligned} E_{x}(V_{t}(x))=\kappa _{t}. \end{aligned}$$

The cost of creating a firm, $\kappa _{t}$, is assumed to grow at rate $g_{e}$.

**Distribution**

The mass of firms in operation born in cohort *j* at time *t* is given by

10 $$\begin{aligned} \eta _{jt}=\mu _{t+1-j}\left( \prod _{s=1}^{j-1}(1-\delta (f_{t-s+1}))\right) \left( 1-F_{t+1-j}(\hat{x}_{jt}/{\tilde{g}_{jt}})\right) , \end{aligned}$$

where $\tilde{g}_{jt}=\prod _{s=1}^{j-1}g_{c,t-s+1}$; and the total mass of firms operating at time *t* is given by

11 $$\begin{aligned} \eta _{t}={\sum _{j=1}^{^{{\infty }}}}\eta _{jt}. \end{aligned}$$

**Dividends**

Firms distribute as dividend to the household all their dividends. The household also needs to finance the start-up costs of newly created firms. Hence, net dividends received from the corporate sector are given by

12 $$\begin{aligned} D_{t}=\sum _{t=0}^{\infty }\left( \mu _{t+1-j}\left( \prod _{s=1}^{j-1}(1-\delta (f_{t-s+1}))\right) \int _{\hat{x}_{jt}}^{\infty }d_{t}(x)dF_{t+1-j}(\hat{x}_{jt}/{\tilde{g}_{jt}})\right) -\mu _{t}\kappa _{t}.\nonumber \\ \end{aligned}$$

**Frictions and main mechanisms**

The model features 4 frictions, which could be technological, policy induced or brought by an exogenous shock: continuation costs *f*, exit spillovers ${{\delta (f)}}$, entry costs $\kappa$ and barriers to technology adoption $\varphi$. Neither of these frictions alters the growth rate of the economy, but they do alter the underlying levels of the BGP, hence generating dynamics in the short and medium run until the economy converges.

*Continuation costs* *f*. Each period, firms need to pay a fixed cost of operation. This could be to due policy frictions, or to an exogenous shock. In this paper, we are going to think of an exogenous shock affecting this economy through a wedge in *f*, $f_{t}=f_{t}^{BGP}(1+\tau _{t});\ \tau _{t}\ge 0$. We are only going to look at transitory shocks to $\tau$ which is mean 0. This implies that suddenly and unexpectedly, it is more costly to operate, so all firms’ profits fall. This shock is especially hurtful for small and low-efficiency firms, since this fixed cost bears a larger weight on firms’ profits.

*Exit spillovers* ${{\delta (f_{t})}}$. We assume that there is an increasing relationship between the cost of operation, $f_{t}$, and exogenous exit. More concretely, we assume the following functional form for this relationship:

13 $$\begin{aligned} \delta _{t}(f_{t})=\theta exp(\phi f_{t}+\vartheta ). \end{aligned}$$

The rationale for this is that while small shocks improve selection, crowding out low-efficiency firms, a large shock can induce further distortions to viable firms, such as input–output disruptions or lack of financing due to macroprudential risks, that are captured in a reduced-form manner by the functional form of 13: for high values of *f*, not only low-efficiency firms exit, but it also increases the exit of productive firms. This functional form will be key to matching the nonlinear relationship between exit rates and TFP growth.

*Entry costs* $\kappa$. Potential entrants need to pay a fixed cost of operation before entering the economy and drawing efficiency *x*, hence preventing firms to enter. Entry costs could be due to technological reasons or policy distortions, affecting the number of firms that decide to enter and their efficiency.

*Barriers to technology adoption* $\varphi _{t}$. Once firms pay the fixed cost of entry, they draw their efficiency from the Pareto distribution in Eq. (8). Hence, the parameter $\varphi _{t}$ characterizes the barriers to technology adoption of firms that want to enter. Whenever $\varphi _{t}>1$, the distribution from which firms draw their efficiencies is stochastically dominated by the frontier efficiency distribution.

### Market clearing conditions

In the closed economy equilibrium, all markets clear. Concretely, we have the following market clearing conditions.

*Labour market clearing condition*. The supply of labour of the household equals the demand of labour of firms. The total amount of labour available, $L_{t}$, is normalized to 1,

14 $$\begin{aligned} L_{t}=1=\sum _{t=0}^{\infty }\left( \mu _{t+1-j}\left( \prod _{s=1}^{j-1}(1-\delta (f_{t-s+1}))\right) \int _{\hat{x}_{jt}}^{\infty }l_{t}(x)dF_{t+1-j}(\hat{x}_{jt}/{\tilde{g}_{jt}})\right) .\nonumber \\ \end{aligned}$$

*Bond market clearing condition*. Bonds are in zero net supply, hence

15 $$\begin{aligned} B_{t}=0. \end{aligned}$$

*Goods market clearing condition*. The good market clears, that is,

16 $$\begin{aligned} Y_{t}= & {} \sum _{t=0}^{\infty }\left( \mu _{t+1-j}\left( \prod _{s=1}^{j-1}(1-\delta (f_{t-s+1}))\right) \int _{\hat{x}_{jt}}^{\infty }xl_{t}(x)^{\alpha }dF_{t+1-j}(\hat{x}_{jt}/{\tilde{g}_{jt}})\right) \nonumber \\ {}= & {} C_{t}+\eta _{t}f_{t}+\mu _{t}\kappa _{t}. \end{aligned}$$

### Balanced growth path

The balanced growth path is an equilibrium along which the sequence of output, consumption, wages, dividends and the efficiency threshold grow at rate $g_{e}-1;$ the mass of potential entrants ${{\eta _{t}}}$ and the mass of active firms ${{\mu _{t}}}$ is constant, and the price of capital is given by ${{q_{t+1}}={\beta /g_{e}}}$.

## Calibration

We calibrate the economy such that it replicates key features of the Spanish economy. The model period is five years. Table 5 shows the main calibration parameters and its targets. First, we externally set some parameters. We make the entry cost to be 0.82 of the continuation cost, following Barseghyan and DiCecio (2011). The parameter $\alpha$ matches the labour share in Spain, which is 0.6 according to Estrada et al. (2012). We set $\beta$ equal to $0.98^{5}$, to match a real interest rate of 2%, and we set the empirical spillover $\epsilon$ to be 0.64 following Asturias et al. (2017).

**Table 5:** Calibration

Parameter		Value	Target	Source
*Exogenously set*				
Entry cost	$\kappa$	0.82*f	Entry cost/continuation cost: 0.82	Barseghyan and DiCecio (2011)
Returns to scale	$\alpha$	0.6	Labour share Spain	Estrada et al. (2012)
Discount factor	$\beta$	0.98	Real interest rate of 2%	Asturias et al (2021)
Spillover term	$\varepsilon$	0.64	Empirical spillover	Asturias et al (2021)
*Internally calibrated*				
Operating cost	$f^{BGP}$	2.900	Average firm size: 14.49	CBI
Tail parameter	$\gamma$	5.004	S.D. of firm size: 247.58	CBI
Firm growth	$g_c$	$1.006^5$	FHK contribution of exit to Spanish productivity growth: 14%	CBI, DIRCE
Entrant efficiency growth	$g_e$	$1.008^5$	Yearly TFP growth rate Spain: 0.53%	CBI, DIRCE
Location parameter $\delta (f)$	$\theta$	12.968	Exit rate: 1.33%	CBI
Shape parameter $\delta (f)$	$\phi$	3.587	Nonlinearity exit-TFP (see graph)	CBI, DIRCE
	$\vartheta$	$-$16.407	Nonlinearity exit-TFP (see graph)	CBI, DIRCE

Internally set targets come from Central de Balances Integrada. The first four targets are for the period 2013–2018. The exit rate and the nonlinearity are computed following Sect. 3 and the methodology explained in the main text of this Section

We calibrate internally in the model the following parameters. We set *f* to 2.9 to match the average firm size (employment) in Spain, 14.49; and we use the tail parameter of the Pareto distribution $\gamma$ to match the standard deviation of firm size, 247.58. We set $g_{e}$ equal to $1.008^{5}$ to match the BGP yearly TFP growth rate of 0.53%^9^; and set $g_{c}$ equal to $1.006^{5}$ to match the contribution of exit to TFP growth in the FHK decomposition, 14%.

As explained before, we assume the following functional form for the exogenous exit rate,

$$\begin{aligned} \delta (f)=\theta exp(\phi f+\vartheta ). \end{aligned}$$

**Fig. 2:**
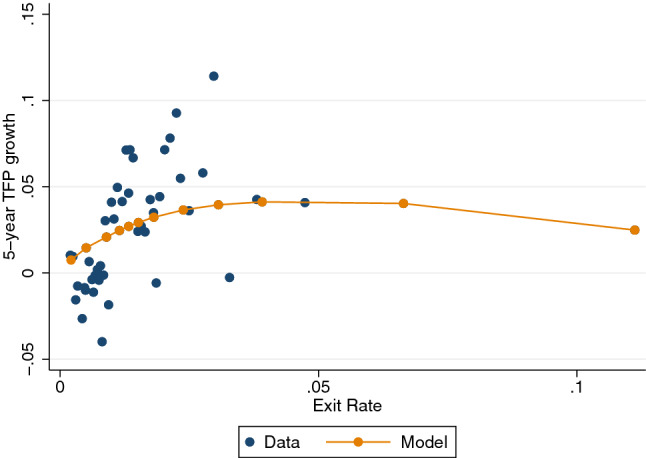
Relationship between exit rates and TFP growth in the model and data. Notes: Source: Central de Balances Integrada and DIRCE. Blue dots are binned sectoral data relating exit rates with TFP growth, using the data used in Sect. 3.2. Orange dots are model outcomes: for exogenous changes in *f*, we plot the endogenous response of exit rates (annualized) and the 5-year TFP growth (see text)

We use the parameter $\theta$ to match the annual exit rate in the BGP, which is 1.33%, and use the shape parameter $\phi$ and location parameter $\vartheta$ to minimize the distance between the model-generated relationship between exit rates and productivity growth and that of the data. To do so, we proceed in the following way. First, compute the value of $\delta ^{*}$ that delivers an annualized exit rate of 1.33% in the BGP, which is the average exit rate in the data, given $f^{BGP}$ from the BGP (that is, $\Delta =0$). Second, guess a value of $\phi$ and $\vartheta$. For a given $\phi$ and $\vartheta$, we obtain the $\theta$ that gives us $\delta ^{*}$. Third, compute the 5-year TFP change in the economy after eight pre-defined shocks $\Delta$ to *f* (that is, $f=(1+\Delta )f^{BGP}$), which delivers an endogenous exit rate.^10^ We calculate a loss function constructed as the difference between the model implied TFP and the data expected TFP for each of the exit rates, targeting a two-piecewise linear approximation of the nonlinearity.^11^ We use a minimization routine to find the parameters $\phi$ and $\gamma$ that minimize these errors.

Figure 2 shows the binned sectoral data points of exit rates and 5-year TFP growth that we use for the calibration, and the orange connected dots show the model outcomes for different changes in *f*. Although there is a lot of dispersion in the data, the model does a relatively good job in matching the nonlinearity pattern in the data. Note the model allows us to make out-of-sample predictions, which can be potentially very useful to understand the dynamics and composition after large and unprecedented shocks.

## Counterfactual exercises

The aim of this section is to perform counterfactual exercises for different scenarios of exit rates that might occur due to the COVID-19 crisis and the subsequent following events.^12^ The way we perform the exercises is as follows: we increase the shock to the fixed cost of operation *f* for one model period (5 years), such that we match the cumulated 5-year exit rate for each scenario. We begin by making assumptions of the exit rate at impact, that is, at year one.

The key issue is how to choose these yearly exit rate scenarios. We use data provided by Central de Balances regarding the level of distressed firms in 2020. We use two measures that are defined by Central de Balances: firms in legal bankruptcy due to losses; and percentage of firms at risk of non-viability.

– *Percentage of firms in legal bankruptcy due to losse*s (empresas en causa legal de disolución por pérdidas). Firms fall into this category if net worth (patrimonio neto) over equity (capital social) is lower than one-half, i.e. networth / (equity/2) $< 1$. An individual firm that fulfils this criterion is in condition to legally file for bankruptcy (causa legal de disolución) according to the law (artículo 363, Ley de Sociedades de Capital).

– *Percentage of firms at risk of non-viability* (empresas en riesgo de inviabilidad). Firms fall into this category if net worth (patrimonio neto) over equity (capital social) is lower than one-half, i.e. networth / (equity/2) $< 1$; and their interest coverage ratio is lower than 1 in the two previous years.

**Fig. 3:**
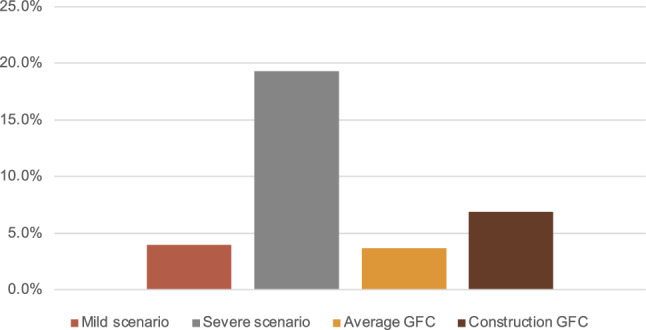
Exit rate scenarios. Notes: Source: Central de Balances and DIRCE. Scenarios based on the percentage of firms in distress in 2020. Mild scenario: percentage of firms at risk of non-viability (red bar). Severe scenario: percentage of firms in legal bankruptcy due to losses (grey bar). For the sake of comparison, we add the exit rate we would have seen in our baseline economy if the increase in exit rate was that of the average exit rate during the economy in the Great Financial crisis (yellow) and that of the most affected sector in the Great Financial crisis, the construction sector (brown)

We present in Fig. 3 these percentages at the aggregate level. The main assumption is that the shock is such that all these firms exit at impact. Note that firms falling within the non-viability definition are firms in profound distress, and this is why we believe that these firms exiting can be thought of as a ‘*mild scenario*’ for exit rates. In the aggregate, if all of them exit, it would imply an increase in the yearly exit rate of 2.7% points, which is close to the increase of 2.4% points increase observed in the aggregate during the Great Recession (in yellow). There are many more firms that fall within the category to fulfil for legal bankruptcy—nearly 20% of all firms. It is because of this reason we think of this as the ‘*severe scenario*’ for exit rates, which would mean an increase in yearly exit rates of 17.7% points, something that has never been experienced in the past, but which might still be feasible due to the magnitude of the COVID-19 and subsequent shock. This scenario would imply an exit rate nearly four times as big as the one in the construction sector during the Great Recession (in brown).

Since the scenarios are going to give the exit rate at impact (year one), we need to make assumptions on the evolution of this exit rate in the following 4 periods after the shock to compute the cumulative 5-year exit rate. We assume that this implicit yearly exit rate goes back to its long-run average with a yearly persistence of 0.88,^13^ and then we cumulate them for 5 years.^14^ Then, we find the increase in *f* that delivers this 5-year exit rate. Note that this increase in the fixed cost of operation increases exit via two margins: first, increasing endogenous exit of less productive firms by increasing the threshold for exiting; and second, increasing the exit spillovers, or the exogenous and random exit of productive firms, that comes from the calibrated function $\delta (f)=\theta exp(\phi f+\vartheta )$. Since exit spillovers increase nonlinearly with the fixed cost of operation, the relative importance of exogenous exit depends on the magnitude of the shock.

**Fig. 4:**
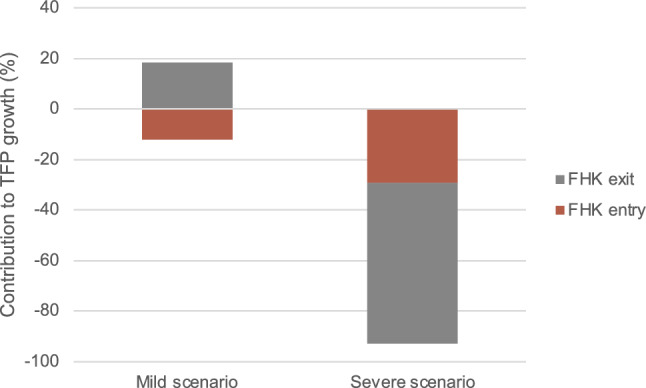
Contribution of exit and entry to TFP growth. Notes: the figure depicts the entry (red) and exit (grey) components of the FHK decomposition, divided by $\Delta$Z and multiplied by 100 to show the contribution in percentages. Model period is 5 years

Table 6 shows the 5-year changes in TFP and the mass of active firms under the mild and the severe shock. The balanced growth path is calibrated such that the 5-year TFP growth is 2.7%. Under the mild shock, the fixed cost of operation increases by 10.25%. TFP growth increases by 1.42 p.p., that is, up to 4.12%. The reason is that when the cost of operation is higher, exit rates increase (see Table 7, column 2), and the mass of active firms decreases 13.8% (Table 6, column 4). However, relatively less efficient firms are the ones that exit the market, so the relative productivity of exiters increases compared to the BGP (see Table 7, column 3), which makes the contribution of exit to TFP growth increase (see Table 7, column 1; and Fig. 4). The exit spillovers are still low, so more productive firms are not forced out of the market. Because this shock decreases the value of starting a firm, there are less firms entering and the contribution of entry to TFP growth decreases (see Fig. 4). On the one hand, with the cost of operation being larger, it is less attractive to create a firm, hence the mass of potential entrants and general equilibrium prices decrease, pushing downwards the efficiency threshold. On the other hand, since there is an increase in the operating costs, only more efficient firms are the ones operating, decreasing the mass of firms of active firms and pushing upwards the efficiency threshold. In the case of the mild shock, the second force dominates, pushing up the efficiency threshold by 5.02% (see Table 7, column 4).

**Table 6:** Aggregate changes

$\Delta$	*f*	$g_Z$	Mass of firms
Mild shock	10.25	1.42	$-$13.80
Severe shock	23.05	$-$0.66	$-$36.22

Notes: Deviations in 5-year period. Values are deviations (in pp) from the BGP, except from the relative productivity of entrants/exiters, which are simple deviations; and the exit threshold, which is in percentage change

**Table 7:** Exit dynamics

	FHK exit	Exit rate	Relative exiter	Exit
			productivity	threshold
Mild shock	0.26	9.59	0.02	5.02
Severe shock	$-$0.38	50.31	0.06	0.64

Deviations in 5-year period. All values are deviations (in pp) from the BGP, except from the relative productivity of entrants/exiters, which are simple deviations

Under the severe shock, the cost of operation increases 23.3%, and TFP growth decreases by 0.66 p.p. from its BGP value. The higher shock induces more exit spillovers, which affect not only the least efficient firms, but also the more efficient ones that are forced out of the market, and increases very significantly exit rates - cumulative 5-year exit rates increase 50.3 p.p., from its BGP value of 6.5%. This huge increase in exit decreases the mass of active firms by 36.2%. The threshold for exiting $\hat{x}$ increases 0.64% (see Table 7, column 4), much less than in the case of the mild shock, and the main reason is that the second force previously explained loses relevance against the first force (even though it still dominates): the decrease in the mass of firms, and especially that of high-efficiency firms due to exit spillovers, decreases significantly equilibrium prices and pushes down the exit threshold. This also makes the relative productivity of exiters compared to that of incumbents to be much higher (see Table 7, column 4). Because of all this, the exit margin is responsible for more than half of the fall in TFP ($-$0.38/$-$0.66). With the shock being larger, there are less incentives for firms to enter, so entry decreases and it contributes negatively to TFP growth (see Fig. 4).

**Fig. 5:**
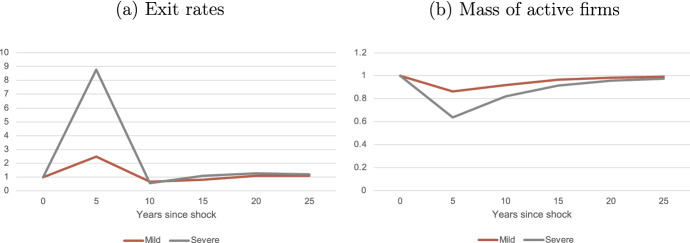
Long-term responses after the shock: exit and the mass of firms. Notes: Outcomes of the model for the mild shock (red); severe shock (grey) and no shock scenario (yellow). Model period is 5 years. All values are normalized to 1. Year 0 (before the shock hits) is the reference year for the normalization. Panel a: exit rates. Panel b: mass of firms

**Fig. 6:**
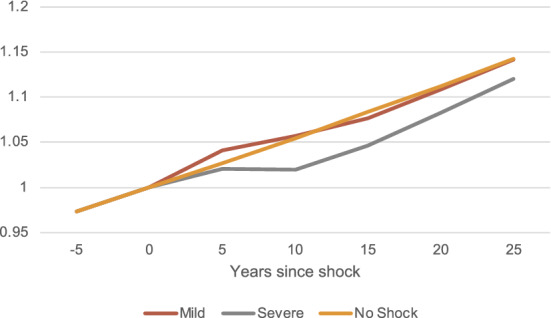
Long-term responses after the shock: TFP. Notes: TFP of the model for the mild shock (red); severe shock (grey) and no shock scenario (yellow). Model period is 5 years. All values are normalized to 1. Year 0 (before the shock hits) is the reference year for the normalization

This shock has effects not only at impact (5 years), but also in the longer run. Note that the growth rates are not affected by the temporary shock, just the underlying BGP level changes, which creates only a temporary deviation from the ’no shock’ scenario. The persistence and depth of the downturn generated depend on the severity of the shock and the scarring effects it generates through the changes in the distribution of firms. Figure 5 shows longer-term dynamics for exit rates and the mass of firms, which are constant along the BGP, and Fig. 6 shows the evolution of TFP, which grows at rate $g_{e}$ along the BGP. They are all normalized to 1 in the year of reference before the shock (year 0). Panel a shows the evolution of exit rates. These increase significantly at impact, to then decrease below its BGP value: when the cost of operation goes back to its initial value, exogenous exit rates decrease. Furthermore, since in the previous period the least efficient firms exited the market, the remaining firms are further from the exit threshold, which also decreases exit. Nonetheless, the mass of active firms in the economy remains significantly below its BGP for nearly 25 years, and the missing mass of firms is significantly larger during all this period in the case of the severe shock.^15^

Figure 6 shows the evolution of TFP when the mild shock hits (red) or the severe shock hits (grey), against the counterfactual scenario of TFP growth when no shock occurs (yellow line). Remember the shock, that is, the increase in the cost of operation, lasts for only one model period. In the case of the mild shock, the medium-term increase in TFP is followed by a decrease in TFP, which falls slightly below the ‘no shock’ scenario before converging back. In the case of the severe shock, TFP decreases even further after the year of impact of the shock, and it takes much longer for TFP to recover: even though entry recovers after the shock, there are still not enough high-efficiency firms in the economy.

Summing up, in this section we have performed different counterfactual scenarios in a model that is consistent with the nonlinear relationship of exit rates and TFP growth observed in the data. The likelihood of each scenario depends on the increase in exit in the aftermath of the COVID-19 crisis, which depends on (a) the severity of the COVID-19 shock itself; (b) the effectiveness of the support policies and the timing when they are lifted; and (c) the subsequent shocks experienced, such as the increase in inflation and the input shortages recently aggravated by the war in Ukraine. In the mild scenario, the increase in exit is moderate and brings a short-term increase in TFP, which speeds up the recovery. In the severe scenario, there is a large increase in exit, not only of low-efficiency firms, but also of high-efficiency firms, which decreases significantly the mass of active firms, and causes a persistent decrease in TFP and a much slower recovery.

## Conclusions

Understanding whether increases in firm exit during crises foster creative destruction, or whether they rather induce scarring effects in the economy is of prime importance both for researchers and for the effective design of the economic policy. We contribute to this debate by reconciling both views. Empirically, using Spanish data we show that increases in exit rates are associated with higher TFP growth when the level of exit is low; in contrast, when exit rates are high, increases in firm exit are associated with decreases in TFP growth. We then build a model of firm dynamics with a balanced growth path following Asturias et al. (2017). This model features three main distortions: a fixed cost of operation, entry costs and barriers to technology adoption. We add a fourth feature, which we denominate exit spillovers: the exogenous exit rate depends on the shock to the fixed cost of operation. Exit spillovers are key to matching the inverted U-shaped relationship between exit rates and TFP. We calibrate the model using Spanish data targeting moments from the firm-size distribution and the Foster et al. (2001) productivity decomposition. We model the COVID-19 shock and the subsequent disruptions that followed it as an increase in the cost of operation of firms that reduces their profitability. In particular, we construct different counterfactual scenarios for this shock to the operation cost that could trigger increases in firm exit according to micro-level information from Central de Balances. As in the data, the relationship between exit rates and TFP growth is nonlinear: if the shock is mild, an increase in exit rates is associated with an increase in TFP growth and a reallocation of resources towards higher efficiency units. However, if the shock is severe, a large increase in exit rates is accompanied by a decrease in TFP growth, since not only low-efficiency firms exit, but also high-efficiency ones, producing scarring effects in the distribution of firms that slow significantly the recovery. Overall, our results stress the importance of keeping exit rates at bay to avoid further scarring effects from the loss of productive firms in the economy that may entail longer and more severe recessions.

## Data Availability

Central de Balances Integrada is openly available upon petition and acceptance in BeLab at (https://doi.org/10.48719/BELab.CIR1621_01). Micro-data from DIRCE are property of INE, and it is not publicly available.

## Appendix

**Table 8:** Cleaning. Remaining observations

	Remaining observations
Total	19,215,517
Drop of excluded sectors or firms without industry	18,320,626
Drop of observations without employment or with incoherent employment	10,422,715
Drop of firms that are not societies	10,371,185
Drop of observations before entry and after exit	9,903,533
Drop of observations that are out of the range 2000–2018 years	9,018,418
Drop of observations where the TFP can’t be computed	6,951,552

This table shows the remaining observations in each step performed in the cleaning procedure

## Appendix A: Cleaning of variables from CBI

We use a similar cleaning as in Albrizio et al. (2021). Our sample data are firms that are active any year from 1996 to 2018. As we indicated in 2, any observation that is younger than the defined entry or older than the defined exit is excluded. There are 71 different economic sectors (CNAE with 2-digits) in the sample. The mining, primary sector, the financial sector, and the public administration are excluded, since they are too small or have a public character that might be cumbersome for our analysis. Each firm has been associated with the industry where that firm has spent most time and when a firm has no industry associated then it is excluded from the sample. Also, we just consider firms that are societies (Sociedad Anónima -SA- or Sociedad Limitada -SL-).

Variables are cleaned by replacing the observations of capital, output, value added and leverage with a missing value when they are negative, also we do this replacement for too high values of leverage (over 10). Concerning employment, just observations in which employment is bigger than 0 and there is coherent employment are kept in the sample. Nominal variables are deflated. The tangible capital is deflated using the 2-digit industry capital investment deflator, whereas the value added and output are deflated with the 2-digit industry value added deflator, then we winsorize these three variables at 1%. Finally, we compute the total factor productivity at the firm level following the methodology proposed by Wooldridge (2009). We use value added for this estimation, and compute TFP using sector-specific elasticities. We drop from the sample any observation with a missing result, then TFP is winsorized at 1% (Table 8).

## Appendix B: Capital and productivity in the model

We follow Asturias et al. (2017) in the way of measuring productivity in the model. Note that we need to define the capital stock of firms to compute TFP. We think of the fixed costs of operation as an investment in the firm. This implies that the stock of capital of a newly created firm is $k_{t}=f_{t}+\kappa _{t}$, and each period the firm invests $f_{t+1}.$ Assuming that capital depreciates each period by $f_{t}-({\kappa _{t+1}-\kappa _{t})}$. This implies that if the firm continues operating, capital next period is given by $k_{t+1}=\kappa _{t+1}+f_{t+1}$.

The productivity of a firm is given by

17 $$\begin{aligned} \log \left[ z_{t}(x)\right] =\log \left[ y_{t}(x)\right] -\alpha _{lt}\log \left[ l_{t}(x)\right] -\alpha _{kt}\log \left[ k_{t}(x)\right] , \end{aligned}$$

where $\alpha _{lt}=w_{t}/Y_{t}$ is the labour share, $\alpha _{kt}=R_{t}K_{t}/Y_{t}$ is the capital share and $R_{t}=1/q_{t}-1+{\delta _{kt}}$ is the rental rate of capital.

## Footnotes

1 See, for example, the article by Carmen Reinhart and Leora Klapper stressing how forbearance policies made it more difficult for banking supervisors to detect the warning signs of rising loan defaults, which can result in a potentially disastrous non-performing loan (NPL) problem (see the article here).

2 We also show that, although the share of exiters seems to change with the cycle (it was higher during the boom, increased further during the crisis, and decreased during the recovery period), the difference in productivity between incumbents and exiters is increasing monotonically in time.

3 According to the DIRCE, a firm may exit (and enter) the market several times throughout the years. Thus, to obtain the exit for each firm, we take the last year of the exits provided for each firm. At this point, the two databases are merged and observations before the entrance and after the exit are dropped.

4 For this analysis, we drop the observations of the sectors with zero exit rate. Besides, we exclude sectors where the growth of the TFP is smaller than $-0,4$ (just two observations) to avoid outliers driving results.

5 These exit rates fall into the highest 5th percentile the of sector-year exit rate distribution.

6 See Appendix 1 for further details about the notion of firm-level capital in this model, and the computation of TFP.

7 In order to ensure endogenous exit in the balanced growth path, we assume that $\bar{g}<g_{e}^{^{1-\varepsilon }}.$

8 This feature of the model makes the economy grow at a rate $g_{e}$, because even though incumbents only grow at rate $g_{c}$, each period the remaining incumbents are more efficient than the previous cohort by a factor of $g_{e}$.

9 That is a 5-year TFP growth of 2.7% like the one observed in Spain for the period 2013–2018 (see Table 2).

10 Note the model gives us the 5-year cumulative exit rate after these predefined increases in *f*. To find the yearly exit rate of the first year, we assume that yearly exit rate goes back to its BGP value following an AR(1) process with a persistence of 0.88 during the 5-year period (this persistence is estimated in the data by estimating the AR(1) process of exit rates at the sector level via OLS). We find the exit rate in the initial period such that, under this assumption, we match the cumulative 5-year exit rate delivered by the model after the change in *f*.

11 In order to compute the data expected TFP, we approximate the relationship between exit rates and TFP using a two-piecewise linear function, where the break is computed from the maximum in 2, rather than using the quadratic specifications in the empirical section, since this functional form allows for a better fit model-data.

12 Since the model period is five years, the shock would be encompassing the ’pure’ COVID-19 shock, the lifting of the policy support and the impact of further macroeconomic disturbances, such as the increase in inflation and the supply problems brought by the war in Ukraine.

13 This persistence is estimated in the data by estimating the AR(1) process of exit rates at the sector level via OLS.

14 That is, we compute the yearly exit rate of years two, three, four and five as $exit\_rate_{t}=\rho *(exit\_rate_{t-1})+(1-\rho )(exit\_rate_{BGP})$. The cumulated 5-year exit rate following the previous assumptions would be 16% in the case of the mild scenario, and 56.9% in the case of the severe scenario. To put these numbers into perspective, during the Great recession, the actual 5-year cumulated rate in the aggregate was 4.63%, and that of the most affected sector—construction—was 19.17%.

15 This is due not only to the increase in exit rates, but also to the decrease in entry rates caused by the shock (not shown).
